# In-Situ Alloy Formation of a WMoTaNbV Refractory Metal High Entropy Alloy by Laser Powder Bed Fusion (PBF-LB/M)

**DOI:** 10.3390/ma14113095

**Published:** 2021-06-04

**Authors:** Florian Huber, Dominic Bartels, Michael Schmidt

**Affiliations:** 1 Institute of Photonic Technologies, Faculty of Engineering, Friedrich-Alexander Universität Erlangen-Nürnberg (FAU), Konrad-Zuse-Straße 3/5, 91052 Erlangen, Germany; dominic.bartels@lpt.uni-erlangen.de (D.B.); sekretariat@lpt.uni-erlangen.de (M.S.); 2 Erlangen Graduate School in Advanced Optical Technologies (SAOT), Friedrich-Alexander Universität Erlangen-Nürnberg, Paul-Gordan-Straße 6, 91052 Erlangen, Germany

**Keywords:** additive manufacturing, laser powder bed fusion, PBF-LB/M, laser beam melting (LBM), in-situ alloy formation, high entropy alloys (HEA), refractory metals

## Abstract

High entropy or multi principal element alloys are a promising and relatively young concept for designing alloys. The idea of creating alloys without a single main alloying element opens up a wide space for possible new alloy compositions. High entropy alloys based on refractory metals such as W, Mo, Ta or Nb are of interest for future high temperature applications e.g., in the aerospace or chemical industry. However, producing refractory metal high entropy alloys by conventional metallurgical methods remains challenging. For this reason, the feasibility of laser-based additive manufacturing of the refractory metal high entropy alloy W_20_Mo_20_Ta_20_Nb_20_V_20_ by laser powder bed fusion (PBF-LB/M) is investigated in the present work. In-situ alloy formation from mixtures of easily available elemental powders is employed to avoid an expensive atomization of pre-alloyed powder. It is shown that PBF-LB/M of W_20_Mo_20_Ta_20_Nb_20_V_20_ is in general possible and that a complete fusion of the powder mixture without a significant number of undissolved particles is achievable by in-situ alloy formation during PBF-LB/M when selecting favorable process parameter combinations. The relative density of the samples with a dimension of 6 × 6 × 6 mm^3^ reaches, in dependence of the PBF-LB/M parameter set, 99.8%. Electron backscatter diffraction (EBSD) and transmission electron microscopy (TEM) measurements confirm the presence of a single bcc-phase. Scanning electron microscopy (SEM) images show a dendritic and/or cellular microstructure that can, to some extent, be controlled by the PBF-LB/M parameters.

## 1. Introduction

High entropy alloys and related compositions are a relatively young concept for designing alloys. The formation of such multi component alloys was reported independently by Cantor et al. [1] and the research group around J.-W. Yeh in 2004 [2,3,4]. In contrast to most conventional alloys, high entropy alloys do not possess a single main alloying element but comprise a composition of multiple principal elements. The exact definition of high entropy alloys varies from source to source. The most common definition includes alloys consisting of at least five elements with a concentration between 5% and 35% each [5]. Other definitions demand a configurational entropy of at least 1.5 R [6]. However, both definitions are overlapping in large parts. Additionally, further criterions like the formation of a single phase solid solution were suggested [7]. These definitions are discussed extensively in recent review papers [7] and books [6,8]. The focus of the present work lies on the alloy W_20_Mo_20_Ta_20_Nb_20_V_20_, which is considered a high entropy alloy in agreement with all these criterions. The majority of publications on high entropy alloys is focused on 3d transition metal alloys based on the CoCrFeMnNi alloy and its derivatives first reported by Cantor et al. [1]. Recent publications demonstrate successful PBF-LB/M of this group of alloys [9,10], investigate the effect of C [11] and N [12] additions, or examine the wear resistance [13]. Besides PBF-LB/M, different additive manufacturing processes such as wire arc additive manufacturing [14], electron beam melting [15,16] or laser liquid phase sintering [17] of 3d transition metal high entropy alloys are subjects of ongoing research. In contrast to that, refractory metal high entropy alloys are by far less investigated [7]. First results on the alloys WMoTaNb and WMoTaNbV were reported by Senkov et al., in 2010 [18] and 2011 [19], respectively. Refractory metal high entropy alloys possess a wide range of material properties depending on the alloy compositions and are especially interesting for high temperature applications [19]. Since the first publications different refractory metal high entropy alloy compositions and alloy design approaches were investigated with the aim to increase e.g., room temperature ductility [20] or to decrease the density of the alloys by replacing heavy elements like Ta and W by lighter elements. Senkov et al. reported a beneficial effect of an Al addition on the mechanical properties of MoNbTaTiZr and HfNbTaTiZr refractory metal high entropy alloys [21], while also reducing the density of the alloy. Also with the aim of decreasing the density, the properties of four alloys of the CrNbTiVZr system are discussed in [22]. In [23] different heat-treatments for modifying the microstructure of Al_0.5_NbTa_0.8_Ti_1.5_V_0.2_Zr with the aim of increasing the ductility at room temperature are reported. A comprehensive overview of current research activities regarding development of refractory metal high entropy alloys can be found in respective review papers [7,24,25].

Despite first investigations, currently only a small fracture of the possible compositions of refractory metal high entropy alloys is investigated. One reason for this is the laborious preparation of samples. Due to the high melting temperatures, the wide range of melting points of refractory metals (e.g., Vanadium at 1910 °C [26] vs. Tungsten at 3422 °C [26]), and the tendency to segregate during solidification, manufacturing of refractory metal high entropy alloys is challenging. Samples are commonly prepared by vacuum arc melting [18,19] or in the case of thin films by magnetron sputtering [27]. Some alloys are also prepared by casting [23] and subsequent homogenization heat treatments with long durations (>>1 day) in a furnace.

Considering this, laser powder bed fusion (PBF-LB/M) is an appealing approach for manufacturing refractory metal high entropy alloys with high throughput. The intensity of modern high power laser beam sources is sufficient to effortlessly melt even tungsten [28]. Furthermore, small meltpool dimensions of a few hundred µm and high cooling rates in the range of 10^6^ K/s [29] during PBF-LB/M are presumed to mitigate segregation of elements during solidification which is of importance with regard to refractory metal high entropy alloys [18]. In addition, by adjusting the process parameters and scan strategies, the energy input can be controlled very precisely. Starting material for PBF-LB/M are metal powders. Novel alloys can be produced either by atomization of pre-alloyed powder or by in-situ alloy formation during PBF-LB/M from mixtures of commonly available elemental powders [30]. The later approach supports material development and high throughput investigations as the desired alloy composition can easily be produced by mixing of powder components without the need of an expensive powder atomization [30]. This, however, requires careful PBF-LB/M process development, since despite successful application of in-situ alloy formation even for high entropy alloys (e.g., [31]), unmolten particles and an inhomogeneous distribution of elements might occur. This is especially crucial if the difference between the melting points of the alloying elements is large, as is the case for refractory metal high entropy alloys [32].

Laser processing of refractory metal high entropy alloys was first demonstrated by Dobbelstein et al., in 2016 [33] by means of directed energy deposition (DED) and in-situ alloy formation. Very comprehensive results of their work are also published in [34]. In contrast to conventional DED, Dobbelstein et al. are not welding continuous weld seams but stack spot welds onto each other to form cylindrical samples, which limits the geometries that can be manufactured. Also, a re-melting step without powder flow is necessary for homogenization of the element distribution and to avoid unmolten particles. Further research on DED of refractory metal high entropy alloys is published by Moorhead et al. [35] or Li et al. [36], who also successfully demonstrated the formation of refractory metal high entropy alloys by DED.

PBF-LB/M of refractory metal high entropy alloys is even less investigated. First results are published by Zhang et al. [37] for the alloy WMoNbTa. However, only small-scale SEM-images and basic corrosion resistance measurements are shown and further research is necessary to understand the effect of the PBF-LB/M processing conditions on the material properties and the homogeneity of the element distribution after in-situ alloy formation. In this context, the aim of the present work is to explore processing of the refectory metal high entropy alloy W_20_Mo_20_Ta_20_Nb_20_V_20_ by PBF-LB/M and to examine the influence of the process parameters on the resulting microstructural properties.

## 2. Materials and Methods

Though there are more recent refractory metal high entropy alloys with superior properties reported in the literature [7], the alloy W_20_Mo_20_Ta_20_Nb_20_V_20_ was selected for this work’s experiments. Since the concept of refractory metal high entropy alloys is comparably young, and the number of possible alloy combinations is vast, there are only single publications on most of the different alloy combinations, thus providing only limited information per alloy. W_20_Mo_20_Ta_20_Nb_20_V_20_ is one of the first refractory metal high entropy alloys developed by Senkov et al. [18] and therefore also one of the best investigated ones. Consequently there are at least a few publications available that allow a comparison with the results and support discussion.

**Figure 1:**
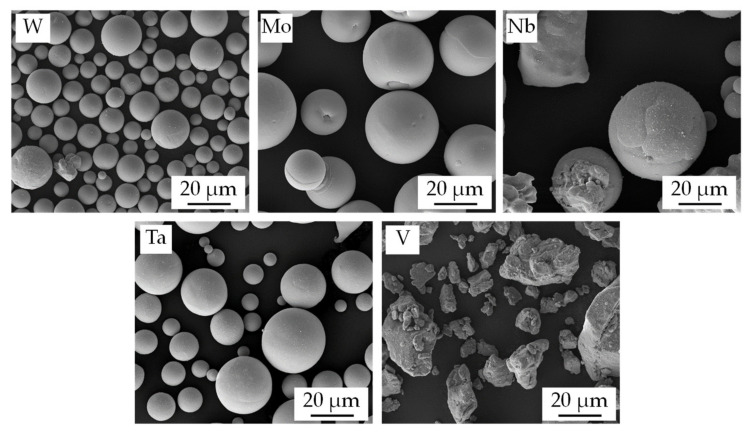
SEM-images of the metal powders used for in-situ alloy formation; W, Mo and Ta powder plasma atomized, Nb powder argon atomized, V powder milled.

For the experiments, a heterogeneous mixture of five single element powders was used to prepare the WMoTaNbV samples. The specifications of the powders used for the PBF-LB/M experiments were chosen based on literature and our own experience. To facilitate PBF-LB/M in the first place, it is mandatory that the powder mixture has sufficient flowability and can be recoated to thin layers [38,39], typically ranging between 20 µm and 100 µm for PBF-LB/M. Furthermore, a low oxygen content of the powders is considered beneficial for manufacturing refractory metal high entropy alloys [34]. Among other factors, the recoatability is determined by particle size and particle shape [38]. Spherical powder particles are in general superior to irregular shaped particles with respect to flowability and are consequently preferable. Also very small particle fractions (<10 µm) should be avoided as they might impair recoatability and bear difficulties with respect to occupational safety [40]. Based on these criteria W-, Mo-, and Ta-powder was purchased from Tekna Plasma Europe SAS (Mâcon, France). The Nb-powder was provided by H.C. Starck Tantalum and Niobium GmbH (Goslar, Germany). Spherical V-powder could unfortunately not be obtained. Hence, milled V-powder from NMD New Materials Development GmbH (Heemsen, Germany) was used. Scanning electron images of the different powder fractions are shown in Figure 1.

All powder particles except the V-powder are predominantly spherical and show a good flowability. The irregular shaped, milled V-powder has a lower flowability than spherical powder. Preliminary recoating test nevertheless proved a sufficient recoatability of the blend for all five powders. The particle size determined by laser diffraction using a Mastersizer 3000 from Malvern Panalytical (Malvern, UK) is listed in Table 1. For the high-melting elements W and Ta a smaller D_90_ (23.1 µm and 36.1 µm respectively) was chosen to ease fusion during PBF-LB/M and to avoid undissolved particles. Since selective evaporation of lower boiling alloying elements is a common phenomenon in PBF-LB/M [41], the amount of Vanadium, which has the lowest boiling point in the WMoNbTaV system [26], in the initial powder mixture was increased by a factor of 1.5 compared to the other alloying elements to pre-compensate the expected evaporation of V during PBF-LB/M. All powders were dried in a vacuum furnace at 120 °C for 8 h and subsequently mixed for 1 h in a Turbular-mix from Willy A. Bachofen AG (Mutenz, Switzerland).

A PBF-LB/M machine of the type AconityMINI (Aconity GmbH, Herzogenrath, Germany) was used for manufacturing cubic samples with an edge length of 6 mm on molybdenum substrate plates obtained from Plansee SE (Reutte, Austria). The machine is equipped with a redPower QUBE single mode fiber laser from SPI Lasers Ltd. (Southamton, UK) featuring an operating wavelength of 1080 nm and a maximum power of 1 kW. The scanner optics used is an AxialScan-30 from Raylase GmbH (Wessling, Germany). The minimal beam diameter on the substrate plate is 70 µm. Argon was used as shielding gas. The oxygen content of the process gas was constantly kept below 25 ppm during PBF-LB/M processing to keep oxygen intake low. PBF-LB/M process parameters were developed experimentally. The following results and discussion section focuses on the two parameter sets described in Table 2. The two parameter sets were chosen based on a preliminary screening study in which we varied laser power, scan speed and hatch distance in a wide range from 100 W to 600 W, 100 mm/s to 1600 mm/s and 45 µm to 180 µm. Both parameter sets were selected for this work because they allow manufacturing of PBF-LB/M samples with high relative densities over 99.5% and represent different areas of the process window with maximum difference in terms of laser power, scan speed and hatch distance to support investigation and discussion of possible effects of the laser parameter set on the material properties. The low laser power/low scan speed parameter set PBF-LB/M B was furthermore derived from previous work on in-situ alloy formation strategies with high melting particles and is assumed to support dissolution of W in the meltpool [32].

The PBF-LB/M samples were separated from the build platform, embedded in epoxy resin, grinded with diamond grinding pads and polished with 3 µm diamond suspensions and oxide polishing suspension with hydrogen peroxide. The preparation depth is in the range of 3 mm and hence in the middle of the 6 mm cubic samples. Samples for transmission electron microscopy (TEM) were subsequently manufactured with a focused ion beam (FIB). The samples were analyzed by optical light microscopy, scanning electron microscopy (SEM), energy dispersive X-ray spectroscopy (EDX), electron backscatter diffraction (EBSD), transmission electron microscopy (TEM) and micro hardness measurements. Relative density values were determined by optical light microscopy and image analysis.

## 3. Results and Discussion

**Figure 2:**
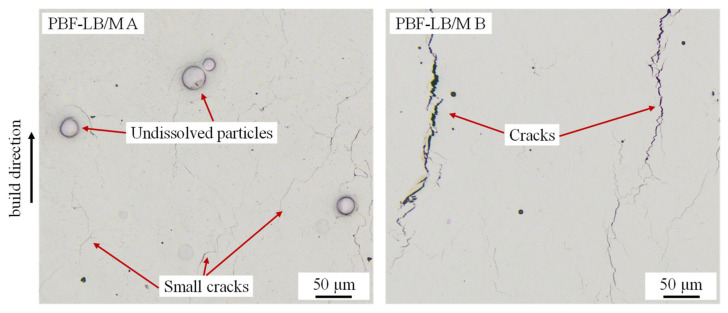
Microsections of WMoNbTaV-samples manufactured with parameter set PBF-LB/M A (**left**) and PBF-LB/M B (**right**).

Figure 2 shows polished microsections of samples built with parameter set PBF-LB/M A and PBF-LB/M B (see Table 2). While the samples exhibit a high relative density of 99.8% in case of parameter set PBF-LB/M A and 99.5% in case of parameter set PBF-LB/M B, both samples still contain defects.

These are essentially cracks, mostly perpendicular to the build direction, and undissolved particles. PBF-LB/M A results in considerably smaller cracks than PBF-LB/M B but contains undissolved powder particles which were identified as tungsten particles by EDS measurements. Tungsten has the highest melting point of the alloy’s elements [26] and consequently is the hardest to dissolve during PBF-LB/M. Undissolved particles are a common phenomenon related to in-situ alloy formation by PBF-LB/M, especially if alloying elements with very different melting points are combined. E.g., Yadroitsev et al. [42] report undissolved Mo particles in Ti-alloys produced by in-situ alloy formation and PBF-LB/M while the lower melting Cu was completely dissolved in the Ti-matrix. Fischer at al. [43] made similar observations with undissolved Nb particles in a Ti matrix. However, they also reported a parameter dependence of the amount of remaining high melting particles. These interdependencies between the PBF-LB/M process parameters and the amount of undissolved high melting particles at in-situ alloy formation are discussed extensively in one of our previous publications [32]. According to [32] a high volumetric energy input, slow scan speeds, and accordingly reduced laser power support the dissolution of high melting elements at in-situ alloy formation by PBF-LB/M. The reasons for this are a higher degree of re-melting, a longer interaction time with the laser beam, and a more favorable powder movement during PBF-LB/M [32]. These relationships apparently also apply for in-situ alloy formation of refractory metal high entropy alloys in the present work. Parameter set PBF-LB/M B comprises a considerably lower scan speed (100 mm/s vs. 800 mm/s) but, despite the lower laser power, a higher volumetric energy density (889 J/mm^3^ vs. 125 J/mm^3^) than parameter set PBF-LB/M A. This facilitates the dissolution of the high melting W particles, thus leading to less undissolved particles as shown in Figure 2. This finding is in good agreement with the results in [32] regarding the dissolution of high melting particles. 

While the sample built with parameter set PBF-LB/M B contains less undissolved particles, parameter set PBF-LB/M A is superior to PBF-LB/M B in terms of cracking. In comparison to parameter set PBF-LB/M B much smaller cracks are apparent in sample PBF-LB/M A. Cracking during PBF-LB/M is a common process-related defect and can have multiple causes. One mechanism, the so called hot cracking, which is related to the solidification of the alloy, is often reported for PBF-LB/M of e.g., Al wrought alloys like the EN AW 6xxx [44] or EN AW 2xxx [45] series. However, based on the crack structure visible in Figure 2, hot cracking is excluded as the main cause of the cracks appearing in WMoTaNbV samples. Instead, cracking of the already solidified material due to internal stress induced by the PBF-LB/M process and an insufficient ductility of the material at lower temperatures is assumed to be the cause for the cracks. According to the results of Zou et al. [46] and Senkov et al. [19] the alloy WMoTaNb possesses only a very limited degree of ductility and fracture toughness at room temperature. While the WMoTaNb grains themselves are comparably crack resistant according to [46], O and N impurities aggregate at grain boundaries and cause embrittlement. This material behavior probably also applies for the alloy WMoTaNbV that is investigated in the present work. The brittle grain boundaries in combination with the internal stress induced during PBF-LB/M consequently lead to cracking of the samples during processing. The different crack patterns in dependence of the parameter set PBF-LB/M A and PBF-LB/M B with wider cracks in the PBF-LB/M B samples are a result of the smaller hatch distance (45 µm vs. 120 µm) and the higher volumetric energy density applied (889 J/mm^3^ vs. 125 J/mm^3^) compared to parameter set PBF-LB/M A. According to the temperature gradient mechanism model that describes the formation of internal stress during PBF-LB/M [47,48] both factors together are assumed to cause higher stress values and consequently wider cracks. Based on these findings, a high temperature (>600 °C) build-platform heating device and/or high purity powder with low O and N content are suggested for crack-free PBF-LB/M of WMoTaNbV. Nevertheless, it is demonstrated that successful in-situ alloy formation of refractory metal high entropy alloys with nearly complete dissolution of the highest melting particles is possible, if favorable parameter combinations are selected. More recently developed refractory metal high entropy alloys like Al_10_Nb_15_Ta_5_Ti_30_Zr_40_ [49] promise an increased room temperature ductility and hence crack-free processing by PBF-LB/M even without high temperature build-platform heating, which needs to verified in future work.

**Figure 3:**
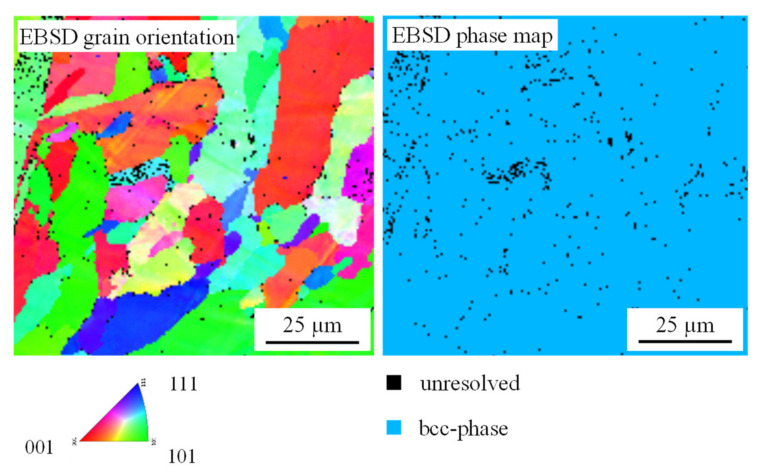
EBSD grain orientation map and EBSD phase map of sample PBF-LB/M B.

The grain and phase structure of a PBF-LB/M B sample was analyzed by EBSD measurements, which are shown in Figure 3. The sample consists entirely of a single bcc high entropy phase, which is in agreement with the results of Senkov et al. for vacuum arc molten WMoTaNbV [18,19]. This confirms the feasibility of producing refractory metal high entropy alloys by PBF-LB/M and in-situ alloy formation. The average grain size determined by the linear intercept method is 16.3 µm. This is more than a factor of four smaller than the 80 µm reported by Senkov et al. for vacuum arc molten samples. The smaller grain size can be attributed to the high cooling rates in the range of 10^6^ K/s during the PBF-LB/M process [50].

**Figure 4:**
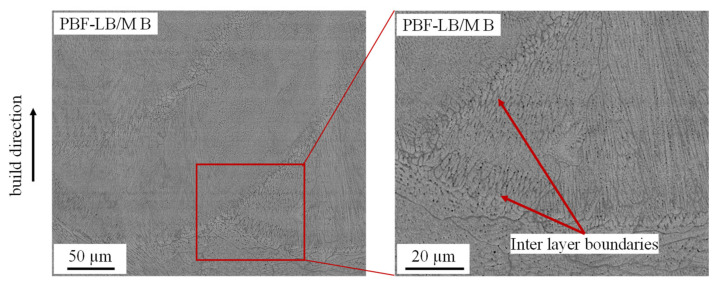
Backscattered electron (BSE) images of sample PBF-LB/M B showing visible melt tracks with coarser structures at the inter layer boundaries.

**Figure 5:**
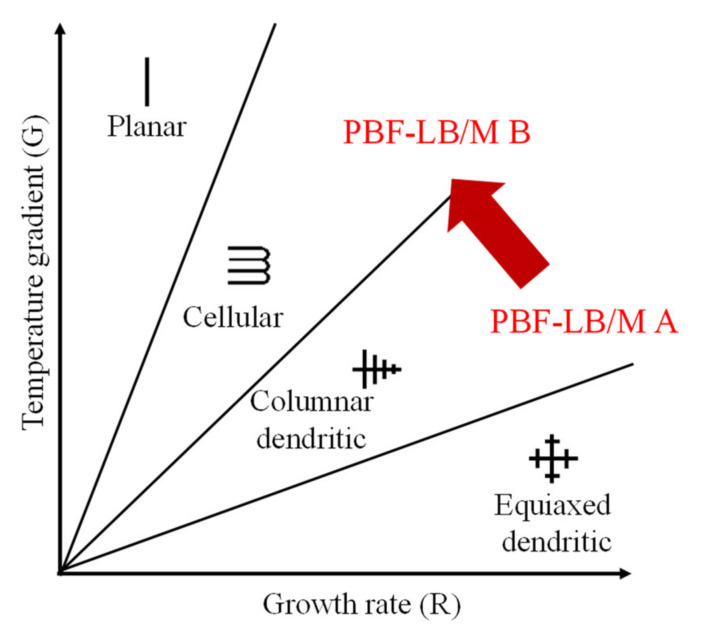
Effect of temperature gradient in the liquid G and growth rate R on the solidification mode and the structure size [52,53].

SEM images reveal an influence of the process parameters and the PBF-LB/M specific solidification conditions on the microstructure. As visible in Figure 4 inter layer boundaries are clearly distinguished by coarser structures. According to [51] temperature gradients in the liquid and growth rates are smaller at the outer contours of the meltpool compared to the inner regions, explaining the coarser structures (see also Figure 5). This demonstrates the sensitivity of the alloy to the solidification conditions and offers the chance to influence material properties by adjusting the PBF-LB/M process conditions/parameters.

This effect is even more evident when comparing the parameter sets PBF-LB/M A and PBF-LB/M B. Based on the process parameters it is safe to assume that the material is subject to higher growth rates R when processed with parameter set PBF-LB/M A compared to parameter set PBF-LB/M B. This is due to the eight times higher scan speed that correlates to some extent with the growth rate [51]. Based on simplified considerations it can further be assumed that the temperature gradient in the liquid is higher for parameter set PBF-LB/M B than for parameter set PBF-LB/M A. The upper temperature of the meltpool is defined by the evaporation temperature of the alloy’s components while the lowest temperature in the meltpool is defined by the solidification range of the alloy. This is equally true for both parameter sets. Considering the differences in the process parameter sets, PBF-LB/M A features larger meltpool dimensions than PBF-LB/M B. Consequently, it is assumed that the temperature gradient in the liquid G is larger for parameter set PBF-LB/M B. According to the literature [52,53] the solidification mode and also the structure size is determined by R and G. While higher growth rates R and lower temperature gradients G in the liquid favor a columnar dendritic solidification, lower growth rates R and higher temperature gradients in the liquid G favor cellular solidification.

**Figure 6:**
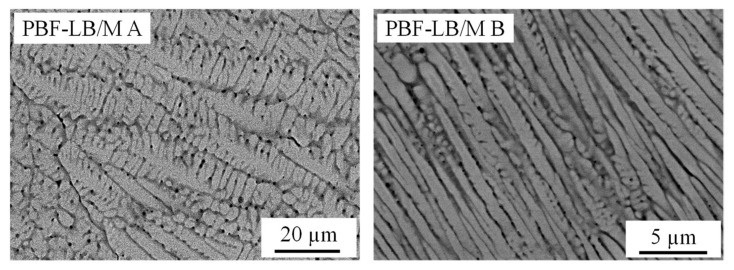
Backscattered electron (BSE) images of the microstructure of sample PBF-LB/M A (**left**) and sample PBF-LB/M B (**right**).

This effect can clearly be seen in Figure 6. Parameter set PBF-LB/M A features a larger structure size and more distinct secondary dendrites, while the material processed with parameter set PBF-LB/M B solidifies in an almost cellular structure with only a few secondary dendrites. These different solidification modes also affect the material properties. The microhardness of the PBF-LB/M A sample with the coarser structure is 561 ± 17 HV0.1 (n = 9) while the microhardness of the PBF-LB/M B sample is 614 ± 21 HV0.1 (n = 9).

**Figure 7:**
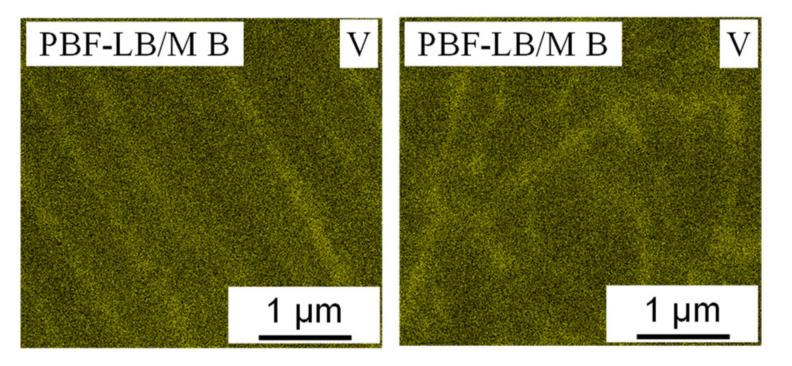
Qualitative EDS maps of the element Vanadium in a PBF-LB/M B sample showing a Vanadium surplus in the interdendrtic areas.

Due to their complex alloy composition and a tendency for segregation, the distribution of the alloy’s elements is of particular interest when it comes to refractory metal high entropy alloys. For this reason, the samples were analyzed by EDS and TEM-EDS respectively. As shown in Figure 7 Vanadium, which is the lowest melting element [54] in the WMoTaNbV alloy, is concentrated in the interdendritic areas, while the intradendritic areas lack Vanadium.

Quantitative EDS point measurements (see Table 3) confirm the vanadium surplus in the interdendritic areas. Nb and Mo are almost equally distributed between inter- and intradendritic regions, while Ta and especially W are predominantly located in the intradendritic areas. These findings are in agreement with results of Senkov et al. [18] who prepared WMoTaNbV samples by vacuum arc melting. Compared to vacuum arc molten samples [18,19] the homogeneity of the element distribution achieved by PBF-LB/M is still very good, which can be attributed to the higher solidification rates and temperature gradients inherent to PBF-LB/M.

**Figure 8:**
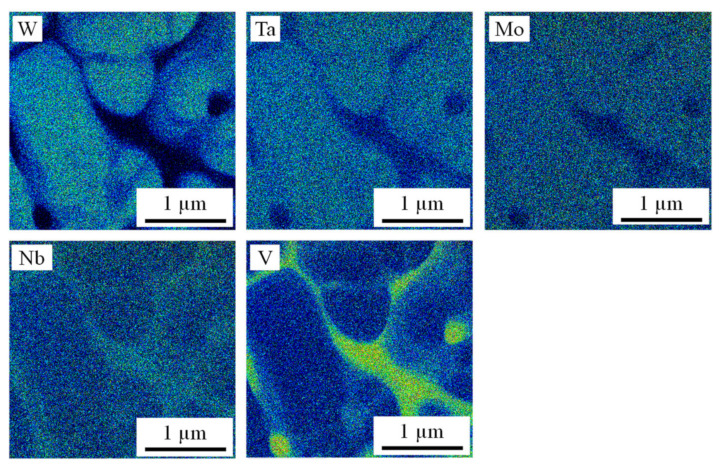
Qualitative TEM-EDS measurements showing the element distribution in a PBF-LB/M A sample.

A PBF-LB/M A sample was analyzed by TEM-EDS with similar results as already observed for parameter set PBF-LB/M B with EDS. Figure 8 shows qualitative TEM-EDS measurements of the element distribution. Mo and Nb are almost equally distributed between interdendritic and intradendritic areas, while there is a considerable W and a weak Ta surplus in the intradendritic regions. V is predominantly concentrated in the interdendritic areas of the material.

## 4. Summary and Conclusions

In the present work, in-situ alloy formation of a WMoTaNbV refractory metal high entropy alloy by PBF-LB/M additive manufacturing is investigated. Samples manufactured with two different parameter sets were analyzed by light and electron microscopy, EBSD, EDS and TEM-EDS. Although high relative densities over 99.8% and a single bcc high entropy phase were achieved, the samples still contained cracks and undissolved W particles. The cracks are a result of the alloy’s low room temperature ductility and can probably be avoided by applying a high temperature heating device or by selecting different alloy compositions with more favorable room temperature properties. It is demonstrated that the number of undissolved high melting particles can be greatly reduced by choosing PBF-LB/M parameter sets with slow scan speeds and accordingly reduced laser power. It is furthermore shown that the PBF-LB/M parameters affect the solidification mode and the resulting microhardness of the alloy. This demonstrates that the alloy WMoTaNbV is sensitive to the processing conditions which are determined by the PBF-LB/M parameters. Besides the mere alloy composition, this opens up opportunities to modify the resulting properties of related additively manufactured refractory metal high entropy alloys. Though, no completely defect free samples were produced the results still demonstrate the general feasibility of in-situ formation of refractory metal high entropy alloys by PBF-LB/M. High cooling rates related to PBF-LB/M mitigate segregation of the elements during solidification without the need of time consuming vacuum arc melting. The possibility of in-situ alloy formation by PBF-LB/M could therefore be a valuable tool to facilitate high throughput investigations that are key to identify application-relevant alloys within the vast number of possible alloy compositions inherent to the high entropy concept—not only for 3d transition metal alloys but also for refractory metal based high entropy alloys.

## Figures and Tables

**Table 1:** Particle size distribution of the powders used for in-situ alloy formation; determined by laser diffraction; mean value with standard deviation from five single measurements.

Powder	D_10_	D_50_	D_90_
W	8.6 ± 0.1 µm	14.0 ± 0.2 µm	23.1 ± 0.5 µm
Mo	19.6 ± 0.3 µm	30.5 ± 0.7 µm	44.3 ± 0.7 µm
Nb	18.7 ± 0.1 µm	35.5 ± 0.4 µm	63.1 ± 0.3 µm
Ta	10.5 ± 0.2 µm	21.0 ± 0.6 µm	36.1 ± 0.7 µm
V	7.8 ± 0.4 µm	24.2 ± 0.1 µm	52.5 ± 1.1 µm

**Table 2:** PBF-LB/M parameter sets used in this work.

Parameter Set	Laser Power	Scan Speed	Spot Diameter	Hatch	Layer Thickness
PBF-LB/M A	600 W	800 mm/s	200 µ	120 µm	50 µm
PBF-LB/M B	200 W	100 mm/s	200 µm	45 µm	50 µm

**Table 3:** Quantitative point measurements in the interdendritic area and the intradendritic area of a PBF-LB/M B sample, respectively; mean value and standard deviation of 6 measurements each.

Position	V	Nb	Mo	Ta	W
Interdentritic at. %	21.8 ± 1.2	20.7 ± 0.4	17.0 ± 0.3	21.1 ± 0.2	15.5 ± 1.3
Intradendritic at. %	11.6 ± 1.6	22.0 ± 0.2	20.1 ± 0.8	23.8 ± 0.5	22.6 ± 0.7
n = 6					

## Data Availability

Not applicable.
